# More Is Always Better Than One: The N-Terminal Domain of the Spike Protein as Another Emerging Target for Hampering the SARS-CoV-2 Attachment to Host Cells

**DOI:** 10.3390/ijms22126462

**Published:** 2021-06-16

**Authors:** Sonia Di Gaetano, Domenica Capasso, Pietro Delre, Luciano Pirone, Michele Saviano, Emilia Pedone, Giuseppe Felice Mangiatordi

**Affiliations:** 1 Institute of Biostructures and Bioimaging, CNR, 80134 Naples, Italy; digaetan@unina.it (S.D.G.); luciano.pirone@cnr.it (L.P.); 2 CIRPEB, University of Naples “Federico II”, 80134 Naples, Italy; domenica.capasso@unina.it (D.C.); michele.saviano@ic.cnr.it (M.S.); 3 CESTEV, University of Naples “Federico II”, 80145 Naples, Italy; 4 Institute of Crystallography, CNR, 70126 Bari, Italy; pietro.delre@ic.cnr.it (P.D.); giuseppe.mangiatordi@ic.cnr.it (G.F.M.); 5 Chemistry Department, University of Bari, 70121 Bari, Italy

**Keywords:** galectin inhibitors, cavity mapping, spike Protein

## Abstract

Although the approved vaccines are proving to be of utmost importance in containing the Coronavirus disease 2019 (COVID-19) threat, they will hardly be resolutive as new severe acute respiratory syndrome coronavirus 2 (SARS-CoV-2, a single-stranded RNA virus) variants might be insensitive to the immune response they induce. In this scenario, developing an effective therapy is still a dire need. Different targets for therapeutic antibodies and diagnostics have been identified, among which the SARS-CoV-2 spike (S) glycoprotein, particularly its receptor-binding domain, has been defined as crucial. In this context, we aim to focus attention also on the role played by the S N-terminal domain (S1-NTD) in the virus attachment, already recognized as a valuable target for neutralizing antibodies, in particular, building on a cavity mapping indicating the presence of two druggable pockets and on the recent literature hypothesizing the presence of a ganglioside-binding domain. In this perspective, we aim at proposing S1-NTD as a putative target for designing small molecules hopefully able to hamper the SARS-CoV-2 attachment to host cells.

## 1. Introduction

The entire world is still living in a great health emergency due to the global pandemic disease COVID-19, caused by the pathogenic SARS-CoV-2 virus. Unfortunately, there are still many questions to be addressed. Although the approved vaccines (https://www.who.int/news-room/q-a-detail/coronavirus-disease-(covid-19)-vaccines) are proving crucial in countering the pandemic threat, they may not be completely effective against new spreading variants.

Vaccines are the most reliable and cost-effective way to avoid and manage infectious diseases [1]. The COVID-19 pandemic and the resulting increase in deaths worldwide have rendered the development of an effective, urgently crucial SARS-CoV-2 vaccine. A worldwide task force carried out the accelerated production and delivery of vaccines to counter the COVID-19 pandemic emergency, which now represent the effective means for terminating the global SARS-CoV-2 pandemic and transforming this infection in a flu-like seasonal illness. Unfortunately, the development of vaccines for SARS-CoV-2 came too late to control the first wave of COVID-19. Although the vaccination campaign is today proceeding quickly and successfully, at least in all the industrialized countries, there is a new emergency due to the insurgence of virus variants that could be able to escape the immune response provided by the vaccines. 

Therefore, developing a therapy to effectively treat SARS-CoV-2 infections is still mandatory. In this context, repurposing known drugs and taking advantage of in-silico approaches are the preferred ways to promptly select clinical candidates [2,3,4,5] within a reasonable timeframe and at acceptable costs. Different examples are available in the literature, mainly based on the application of Virtual Screening (VS) procedures [6]. Of note are research articles that have reported data properly validated by in-silico protocols (e.g., docking calibration) and/or in vitro assays for the identification of: (i) inhibitors of the SARS-CoV-2 main and papain-like proteases [3,4,7]; (ii) compounds targeting the receptor-binding domain (RBD) of the spike SARS-CoV-2 protein [5,8]. 

Indeed, RBD is known to be directly involved in the interaction with the well-known receptor ACE2, which plays a relevant role in the attachment of the virus [9]. In particular, RBD has been identified as the region binding to ACE2 on the cell surface, therefore it has become an attractive target for developing neutralizing antibodies, viral inhibitors, and vaccines [9] aimed at blocking SARS-CoV-2 infection. A study by Tai W. et al. [10] reported a recombinant RBD protein able to bind to ACE2 receptors, thus inhibiting SARS-CoV-2 infection in cells. Trezza et al. [11] identified, by combining docking and molecular dynamics (MD) simulations, lumacaftor and simeprevir as putative spike protein ACE2 interaction inhibitors. Interestingly, the latter was also identified by Kadioglu et al. [12]. Another approach was used by Shehroz et al. [13]: the authors considered the RBD, conserved in all the different 483 SARS-CoV-2 S protein sequences taken into consideration, as a pharmacophore, and identified 1327 lead compounds, of which eight fit the criteria for safe oral drugs. Of mention is also the paper by Cao et al. [14]: building on computer-generated scaffolds based on the ACE2 helix that interacts with RBD or docked against the RBD, the authors designed miniproteins with affinities ranging from 100 picomolar to 10 nanomolar, able to block SARS-CoV-2 infection in Vero E6 cells. 

Notably, more recent studies indicate two other receptors involved in the SARS-CoV-2 attachment [8]: a transmembrane glycoprotein, CD147, localized on the surface of the host cell, and a chaperone heat shock protein, GRP78 [15,16,17]. Both could interact with several ligands as well as the RBD of the viral S protein, acting as multifunctional receptors for viral entry [8]. It is increasingly evident that many receptors are involved in the virus entry, and a recent study carried out by Singh M. et al. [18], using a single-cell RNA expression map approach, identified unequivocally 28 SARS-CoV-2 and coronavirus-associated receptors and factors (SCARFs). In particular, the possible involvement of CLEC4G and CLEC4M in the recognition of the virus glycosylation shield was very recently investigated by Lu Q. et al. [19], who demonstrated, for example, that the N165Q mutation greatly enhanced binding to CLEC4G and CLEC4M but not to ACE2.

**Figure 1:**
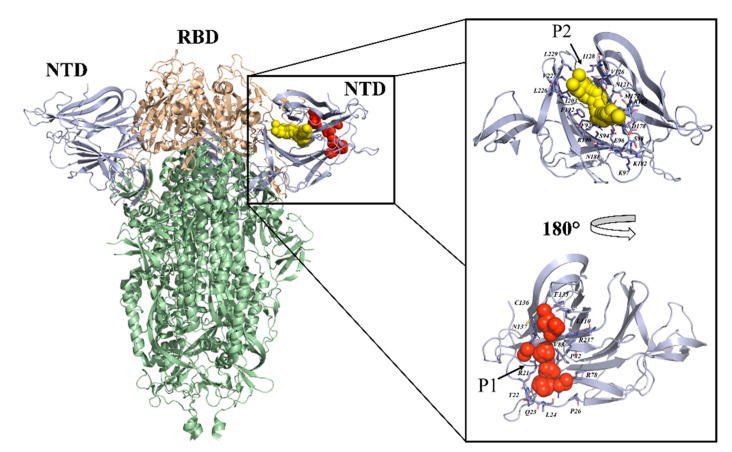
Cryo-EM structure of the SARS-CoV-2 spike (S) glycoprotein (PDB code: 7JJI [42]) employed for the performed cavity mapping. The protein is rendered as cartoon, important residues forming the druggable cavities P1 (red spheres) and P2 (yellow spheres) identified by SiteMap [41] are rendered as sticks in the zoomed inset.

In addition, GPR78 has previously been reported to mediate viral entry of the Ebola virus, Zika virus, influenza virus, HCV, and MERS-CoV [20]. Thus, similar to MERS-CoV, the SARS-CoV-2 S protein could recognize and bind to the GRP78, facilitating viral entry. Recently, a deep characterization [21] of the glycan structures of the RBD from SARS-CoV-2 has been carried out using NMR spectroscopy. In particular, the interaction of RBD with labeled glycans from different lectins has been analyzed. Previous work reported the specific glycosylation pattern [22] in the S protein, identifying 22 N-linked glycosylation sites, among which 52% were fucosylated, and 15% of the glycans contained at least one sialic acid residue. In particular, the two glycosylated residues in the RBD, N331 and N343, played a relevant role in the interaction with lectins. NMR analysis allowed the characterization of the specific glycan epitopes recognized by each lectin, and paves the basis to unveil the roles played by glycosylation patterns in the interaction with receptors during infection. Another paper suggests that RBD can be put into relation with lectins, as a red-alga-derived lectin, griffithsin (GRFT), inhibits SARS-CoV-2 infection by targeting the glycosylation sites in the RBD of the SARS-CoV-2 S protein [23,24]. Although of interest, all these studies border their attention on the RBD of the S protein, despite the evidence that its extracellular portion is also constituted by the N-terminal domain (Figure 1—S1-NTD) encompassing residues 20–286. Importantly, a recent and growing literature also indicates this region as a promising target for both therapeutic and vaccine strategies [25,26,27,28]. To strengthen this hypothesis, recent data showed that several neutralizing antibodies selectively bind to the S1-NTD, hampering the interaction with the host cells [25,28,29]. Surprisingly, MD simulations showed that S1-NTD targeting through small molecules can disrupt interactions between RBD and ACE-2 [30].

Targeting the NTD region as a pharmacological target may be a winning strategy to hinder the attack of SARS-CoV-2 on host cells, also based on the evidence that the mutations detected in the variants identified so far (from the South African variant to the Indian variant etc.) are all picked up in the RBD region. Thus, targeting NTD could also be effective against variants of the virus.

Based on this evidence and background, new drugs can be developed by specifically interacting with the S1-NTD. In particular, in this perspective, we aim to prompt the scientific community to take advantage of new binding sites localized in the NTD of the SARS-CoV-2 S protein to speed up the identification of small molecules able to hamper the SARS-CoV-2 attachment to host cells.

## 2. Results

### 2.1. Targeting S1-NTD with Small Molecules 

#### 2.1.1. Repurposing Sialic Acid Analogues 

Sialic acids have been defined as peculiar for binding several pathogens and toxins. Supported by their location and ubiquitous distribution, they are able to mediate or modulate a wide variety of physiological and pathological processes [31]. In such contexts, different human coronaviruses such as MERS-CoV, HCoV-OC43, and HCoV-HKU1 use alternative strategies, behind the interaction with ACE2, to infect human host cells. In particular, while HCoV-OC43 and HCoV-HKU1 use host sialosides as the sole receptor to infect host cells, MERS-CoV utilizes a dual-receptor strategy binding with both human dipeptidyl peptidase-4 (DPP4) host protein receptor and host sialosides [32,33]. For SARS-CoV-2, several “hiding” sites for recognizing and binding glycans containing sialic acid were recently predicted to be located in the NTD of the S protein [34,35]. In particular, by viral evolution and adaptation processes, distinct binding residues on the NTD allowed getting different ligand specificities, such as 9-O-Ac-sialosides versus non-O-acetylated-sialoglycans. To deeply understand and justify the higher infectivity of SARS-CoV-2 and its ability to use human sialosides as an alternate receptor, a structural analysis of the NTD of SARS-CoV-2 in comparison with that of MERS-CoV and SARS-CoV was carried out, highlighting the presence of three divergent loop regions [32]. Further collected data demonstrated the involvement of these loop regions in the formation of a potential sialoside binding pocket, resembling the one identified in the MERS-CoV S protein and formed by the residues L18-Q23, H66-T78, and G252-S254. This hypothesis has been supported by Milanetti et al. [36] through surface iso-electron density mapping. Building on these studies, this region of the SARS-CoV-2 S protein, similar to that of MERS-CoV but absent in SARS-CoV, could be able to mediate a low-affinity but high-avidity interaction with sialic acid. 

Another approach that identified putative sialoside binding pockets within S1-NTD was used by Behloul et al. [37]. More specifically, the authors, by comparing the structural characteristics of the S1-NTD from SARS-CoV-2 with those from SARS-CoV, identified three insertions, present in SARS-CoV-2 and previously identified in another coronavirus named BCoV [38,39], as forming a binding pocket able to bind sugars such as Neu5,9Ac2. These sugars showed the highest affinity for BCoV NTD, as determined by a glycan sheet array composed of 611 different natural and synthetic mammalian glycans. The pocket includes E154, F157, Y160 and the so-called stabilizing loop (N122-N125). A different sialoside binding pocket in the S1-NTD was suggested by Baker et al. [40] as a result of a sequence alignment among other coronavirus S proteins, especially against the known sialic binding protein from HCOV-OC43. The authors not only identified a potential sialic acids binding site, where the involved residues (R21, Q23, L24, H69, F79, P82, and R246) seem not particularly conserved, apart from F79 and P82, but also demonstrated its glycan-binding property, employing a glyconanoparticle platform [40]. 

In order to assess whether the sialoside binding pockets hypothesized in the literature might represent a valuable starting point for repurposing small molecules (e.g., sialic acid analogues), we performed a druggability assessment of all the putative ligand-binding pockets within the S1-NTD. In particular, SiteMap [41], available from the Schrodinger suite 2019-4, was used as software, and a recent deposited cryo-EM structure of the S-protein (PDB code: 7JJI) [42] was employed as a 3D protein model (Figure 1). Notice that such a model was selected, among the several S1-NTD structures available in The Protein Data Bank, since it includes all the S1-NTD residues (starting from Q14). SiteMap allows the computation of two scores for each predicted cavity: (i) SiteScore, based on the cavity size, degree of enclosure, and hydrophobicity, and (ii) DScore, for assessing cavity druggability. Among the three sialoside binding regions hypothesized in the literature, only that proposed by Baker et al. [40] returns a cavity predicted as probably druggable (Figure 1), the computed SiteScore and DScore being equal to 0.913 and 0.903, respectively [43]. Based on these data, this pocket (hereinafter referred to as P1—Figure 1), including R21, T22, Q23, L24, P26, R78, P82, V83, L110, F135, C136, N137, and R237, might represent a promising target for a further structure-based investigation aimed at repurposing known sialic acid analogues for counteracting SARS-CoV-2 attachment to host cells.

#### 2.1.2. Repurposing Galectin Inhibitors 

In addition to the mentioned sialoside binding pockets, other sites of the S1-NTD have been proposed as potentially of interest for designing small molecules. In particular, a new type of ganglioside-binding domain (GBD) has been recently proposed by Fantini et al. [44]: a strong interaction between GM1 Ganglioside and S1-NTD was suggested based on MD simulations, thus supporting the robustness of a dual attachment model for SARS-CoV-2, as observed in MERS-CoV [25]. Fantini et al. [45] demonstrated the competitive action of hydroxychloroquine (CLQ-OH), a drug already used in COVID-19 therapy, for the ganglioside site. In addition, the same authors, using MD simulations, showed that CLQ-OH /azithromycin (ATM), a combined therapy in use, act in synergy to prevent interaction between the virus and the host cells. Altogether, these data show that ATM is directed against the virus, while CLQ-OH against cellular attachment cofactors [25]. However, the use of CLQ-OH remains controversial as many clinical trials have shown non-significant improvement in patient status, thus not at all supporting the use of this drug for the treatment of COVID-19 among hospitalized adults [46,47]. On the other hand, while the mechanism of ATM in preventing bacterial infections is well-known and indirectly supported by previous experience with other viral pneumonias, chronic lung diseases, and inflammatory disorders [48], its use is not yet accepted in the treatment of COVID-19 due to lack of good quality clinical data.

Significant analogies between Murine hepatitis virus (MHV) S1-NTD and BCoV S1-NTD with the human gal-3 [38,49,50], suggested a functional similarity. Gal-3 belongs to galectins, a large family of glycan-binding proteins with a preference for β-galactoside- containing structures [51], implicated in many diseases, electing them as a relevant target for drug discovery. Several studies have identified a “galectin fold” in NTDs of different coronaviruses [37,52]. The similarity between the structures of gal-3 and S1-NTDs of different betacoronaviridae lets us hypothesize that a galectin gene in their genome was incorporated at a certain time in their evolution [49]. In particular, a high degree of structural similarity was showed comparing the S1-NTD and gal-3 [37,52]. Based on this evidence, it is reasonable to postulate that binding to sugars could also involve the galectin fold, considering the strict structural analogy and the stringent link between galectins and infection. Indeed, it is well-known that galectins play pivotal roles in host–pathogen interactions, such as adhesion of pathogens to host cells, and activation of host innate and adaptive immunity [53]. Considering the high degree of structural and sequence similarity (12%) between S1-NTD and gal-3, it may be possible that existing gal-3 inhibitors, able to modulate the interaction with a sugar, could bind the S1-NTD and could represent a further suitable therapeutic application. Moreover, galectins were proved to activate the pro-inflammatory transcription factor N-f-kB and to induce the release of IL-6 and TNF-a [49]; in addition, an increasing number of patients suffering from COVID-19 showed highly elevated levels of gal-3 together with the so-called “cytokine storm syndrome” (CSS). It is worth mentioning that galectin inhibitors should hold a double effect [37,49], one linked to their anti-inflammatory properties, the other connected with the capacity to bind S1-NTD [49]. During the last few months, several papers reported on the use of galectin inhibitors for the treatment of COVID-19 [39,44,52,54], but it is not clear where such inhibitors could interfere and how they could exert their effect. Sethi et al. [54] indicated, among 330 galectin inhibitors screened, that TD-139, a molecule currently in Phase IIb clinical trials, is able to bind to RBD in the region interacting with ACE2, based on molecular docking and MD simulations. A doubt arises spontaneously: TD139 is a well-known galectin inhibitor and its interaction with the galectin fold has been well characterized [55] by ITC, X-ray crystallography, and NMR studies. Therefore, galectin inhibitors, like TD-139, could interact with the RBD region, but also with the galectin fold, their natural interaction site. In addition, three glycosylation sites were identified in the galectin fold (N122, N149, and N165) [22], suggesting that the interaction could happen also between the glycans on the NTD and gal-3 on the host. Nevertheless, among galectin inhibitors, glycomimetics, structurally and functionally miming carbohydrates but endowed with improved pharmacological properties [51], could represent promising drug candidates. Developing selective glycomimetics is, however, very challenging due to the highly conserved carbohydrate receptor domains (CRDs) in mammalian galectins. Most of the molecules synthesized to date are based on sugar scaffolds, although some non-saccharide-based compounds, as peptide-based mimetics, have also been reported [39,56], opening a new scenario for the design of novel galectin inhibitors for therapeutic applications.

#### 2.1.3. Designing Small Molecules Targeting a New Druggable Cavity

Besides the putative sialoside binding site described in the section titled “Repourposing sialic acid analogues”, the performed cavity mapping revealed the presence of an unexpected cavity within S1-NTD. Notice that such a pocket is localized in a different region with respect to those hypothesized in the literature as putative sialoside binding sites, and, for this reason, is herein proposed as able to efficiently accommodate small molecules designed ad hoc rather than for drug repurposing approaches. More specifically, the predicted cavity (hereinafter referred to as P2—Figure 1) is formed by F92, S94, E96, K97, S98, R102, N121, V126, I128, M177, D178, K182, N188, R190, F192, I203, L226, V227, and L229. Interestingly, P2 is responsible for a SiteScore > 1.000 (1.023) which is typical of sites of particular interest for setting up structure-based drug design approaches. It should be noted that the SiteScore is calibrated so that the average value for a large set of submicromolar sites used as reference is 1.000 [39]. Importantly, P2 also returns a very high Dscore (1.039), which is indicative of its very high druggability [43]. These data, taken as a whole, are in agreement with recent experimental findings. In particular, Bangaru et al. [42] recently published on Science a cryo-EM analysis of a full-length SARS-Cov-2 S protein, revealing the presence of a cavity in the S1-NTD, corresponding to the herein hypothesized P2, able to bind the used detergent (polysorbate 80) by establishing H-bond interactions involving R190 and H207. The authors concluded that such a pocket can be considered a “potential target for drug design against SARS-CoV-2”. Remarkably, while the present paper was under review, Rosa et al. [57] showed, using cryo-electron microscopy and X-ray crystallography, that two small molecules (i.e., the products of heme metabolism biliverdin and bilirubin) target the SARS-CoV-2 S protein with nanomolar affinity by interacting with P2. In summary, the computed size, degree of enclosure, hydrophobicity, and druggability, combined with some recent experimental observations, put forward P2 as a relevant site for further structure-based drug discovery approaches to be followed to identify new small molecules able to bind the SARS-CoV-2 S protein. 

## 3. Conclusions

The S glycoprotein plays a crucial role in SARS-CoV-2 attachment to the host cells and, for this reason, has been the object of intensive research efforts in the last few months, mainly focused on the protein portion (i.e., the RBD) responsible for binding with the well-known receptor ACE2. In this paper, we prompt the scientific community to also consider the NTD of the protein as emerging experimental pieces of evidence, supported by in-silico data, suggest this protein portion to be worth investigation to identify and/or design small molecules for new therapeutic strategies against COVID-19. 

In particular, by combining a computational detection of druggable cavities with shreds of evidence from the recent literature, we propose three different S1-NTD sites to be further investigated: (i) a sialoside binding site hypothesized by combining computational data with recently published experimental findings; (ii) a ganglioside binding domain inside a galectin-fold based on experimental pieces of evidence reported in the literature, and (iii) a new cavity predicted by SiteMap as particularly suited for setting up structure-based drug design approaches. As further evidence for galectin-3 involvement, a very recent article should be cited reporting that a galectin-3 binding protein (LGALS3BP) was identified as an interaction partner of the SARS-CoV-2 spike glycoprotein [58]. Overexpression of LGALS3BP inhibits spike pseudoparticle uptake and spike-induced cell–cell fusion in vitro. Although further work is required to experimentally validate the discussed in silico predictions, the perspective provides a valuable starting point for medicinal chemists and structural biologists interested in this field. 

## Data Availability

Data sharing not applicable.

## References

[1] Awadasseid A., Wu Y., Tanaka Y., Zhang W. (2021). Current advances in the development of SARS-CoV-2 vaccines. Int. J. Biol. Sci..

[2] Verma A.K., Aggarwal R. (2021). Repurposing potential of FDA-approved and investigational drugs for COVID-19 targeting SARS-CoV-2 spike and main protease and validation by machine learning algorithm. Chem. Biol. Drug Des..

[3] Augustin T.L., Hajbabaie R., Harper M.T., Rahman T. (2020). Novel Small-Molecule Scaffolds as Candidates against the SARS Coronavirus 2 Main Protease: A Fragment-Guided in Silico Approach. Mol. Basel Switz..

[4] DelRe P., Caporuscio F., Saviano M., Mangiatordi G.F. (2020). Repurposing Known Drugs as Covalent and Non-covalent Inhibitors of the SARS-CoV-2 Papain-Like Protease. Front. Chem..

[5] Carino A., Moraca F., Fiorillo B., Marchianò S., Sepe V., Biagioli M., Finamore C., Bozza S., Francisci D., Distrutti E. (2020). Hijacking SARS-CoV-2/ACE2 Receptor Interaction by Natural and Semi-synthetic Steroidal Agents Acting on Functional Pockets on the Receptor Binding Domain. Front. Chem..

[6] Passeri G.I., Trisciuzzi D., Alberga D., Siragusa L., Leonetti F., Mangiatordi G.F., Nicolotti O., International Management Association (2020). Strategies of Virtual Screening in Medicinal Chemistry. Data Analytics in Medicine: Concepts, Methodologies, Tools, and Applications.

[7] Sardanelli A., Isgrò C., Palese L. (2021). SARS-CoV-2 Main Protease Active Site Ligands in the Human Metabolome. Molecules.

[8] Tito A., Colantuono A., Pirone L., Pedone E., Intartaglia D., Giamundo G., Conte I., Vitaglione P., Apone F. (2021). Pomegranate Peel Extract as an Inhibitor of SARS-CoV-2 Spike Binding to Human ACE2 Receptor (in vitro): A Promising Source of Novel Antiviral Drugs. Front. Chem..

[9] Mercurio I., Tragni V., Busto F., De Grassi A., Pierri C.L. (2021). Protein structure analysis of the interactions between SARS-CoV-2 spike protein and the human ACE2 receptor: From conformational changes to novel neutralizing antibodies. Cell. Mol. Life Sci..

[10] Tai W., He L., Zhang X., Pu J., Voronin D., Jiang S., Zhou Y., Du L. (2020). Characterization of the receptor-binding domain (RBD) of 2019 novel coronavirus: Implication for development of RBD protein as a viral attachment inhibitor and vaccine. Cell. Mol. Immunol..

[11] Trezza A., Iovinelli D., Santucci A., Prischi F., Spiga O. (2020). An integrated drug repurposing strategy for the rapid identification of potential SARS-CoV-2 viral inhibitors. Sci. Rep..

[12] Kadioglu O., Saeed M., Greten H.J., Efferth T. (2021). Identification of novel compounds against three targets of SARS CoV-2 coronavirus by combined virtual screening and supervised machine learning. Comput. Biol. Med..

[13] Shehroz M., Zaheer T., Hussain T. (2020). Computer-aided drug design against spike glycoprotein of SARS-CoV-2 to aid COVID-19 treatment. Heliyon.

[14] Cao L., Goreshnik I., Coventry B., Case J.B., Miller L., Kozodoy L., Chen R.E., Carter L., Walls A.C., Park Y.-J. (2020). De novo design of picomolar SARS-CoV-2 miniprotein inhibitors. Science.

[15] Carlos A.J., Ha D.P., Yeh D.-W., Van Krieken R., Tseng C.-C., Zhang P., Gill P., Machida K., Lee A.S. (2021). The chaperone GRP78 is a host auxiliary factor for SARS-CoV-2 and GRP78 depleting antibody blocks viral entry and infection. J. Biol. Chem..

[16] Ibrahim I.M., Abdelmalek D.H., Elshahat M.E., Elfiky A.A. (2020). COVID-19 spike-host cell receptor GRP78 binding site prediction. J. Infect..

[17] Smaldone G., Pirone L., Capolupo A., Vitagliano L., Monti M.C., Di Gaetano S., Pedone E. (2018). The essential player in adipogenesis GRP78 is a novel KCTD15 interactor. Int. J. Biol. Macromol..

[18] Singh M., Bansal V., Feschotte C. (2020). A Single-Cell RNA Expression Map of Human Coronavirus Entry Factors. Cell Rep..

[19] Lu Q., Liu J., Zhao S., Castro M.F.G., Laurent-Rolle M., Dong J., Ran X., Damani-Yokota P., Tang H., Karakousi T. (2021). SARS-CoV-2 exacerbates proinflammatory responses in myeloid cells through C-type lectin receptors and Tweety family member 2. Immunity.

[20] Ibrahim I.M., Abdelmalek D.H., Elfiky A.A. (2019). GRP78: A cell’s response to stress. Life Sci..

[21] Lenza M.P., Oyenarte I., Diercks T., Quintana J.I., Gimeno A., Coelho H., Diniz A., Peccati F., Delgado S., Bosch A. (2020). Structural Characterization of N-Linked Glycans in the Receptor Binding Domain of the SARS-CoV-2 Spike Protein and their Interactions with Human Lectins. Angew. Chem. Int. Ed..

[22] Watanabe Y., Allen J.D., Wrapp D., McLellan J.S., Crispin M. (2020). Site-specific glycan analysis of the SARS-CoV-2 spike. Science.

[23] Kurhade S.E., Weiner J.D., Gao F.P., Farrell M.P. (2021). Functionalized High Mannose-Specific Lectins for the Discovery of Type I Mannosidase Inhibitors. Angew. Chem. Int. Ed..

[24] Cai Y., Xu W., Gu C., Cai X., Qu D., Lu L., Xie Y., Jiang S. (2020). Griffithsin with A Broad-Spectrum Antiviral Activity by Binding Glycans in Viral Glycoprotein Exhibits Strong Synergistic Effect in Combination with A Pan-Coronavirus Fusion Inhibitor Targeting SARS-CoV-2 Spike S2 Subunit. Virol. Sin..

[25] Fantini J., Chahinian H., Yahi N. (2021). Leveraging coronavirus binding to gangliosides for innovative vaccine and therapeutic strategies against COVID-19. Biochem. Biophys. Res. Commun..

[26] Li Y., Wang T., Zhang J., Shao B., Gong H., Wang Y., Liu S., Liu T.-Y. (2021). Exploring the Regulatory Function of the N-Terminal Domain of SARS-CoV-2 Spike Protein Through Molecular Dynamics Simulation. arXiv.

[27] McCallum M., De Marco A., Lempp F.A., Tortorici M.A., Pinto D., Walls A.C., Beltramello M., Chen A., Liu Z., Zatta F. (2021). N-terminal domain antigenic mapping reveals a site of vulnerability for SARS-CoV-2. Cell.

[28] Suryadevara N., Shrihari S., Gilchuk P., VanBlargan L.A., Binshtein E., Zost S.J., Nargi R.S., Sutton R.E., Winkler E.S., Chen E.C. (2021). Neutralizing and protective human monoclonal antibodies recognizing the N-terminal domain of the SARS-CoV-2 spike protein. Cell.

[29] Cerutti G., Guo Y., Zhou T., Gorman J., Lee M., Rapp M., Reddem E.R., Yu J., Bahna F., Bimela J. (2021). Potent SARS-CoV-2 neutralizing antibodies directed against spike N-terminal domain target a single supersite. Cell Host Microbe.

[30] Olotu F.A., Omolabi K.F., Soliman M.E. (2020). Leaving no stone unturned: Allosteric targeting of SARS-CoV-2 spike protein at putative druggable sites disrupts human angiotensin-converting enzyme interactions at the receptor binding domain. Inform. Med. Unlocked.

[31] Varki A. (2008). Sialic acids in human health and disease. Trends Mol. Med..

[32] Park Y.-J., Walls A.C., Wang Z., Sauer M.M., Li W., Tortorici M.A., Bosch B.-J., DiMaio F., Veesler D. (2019). Structures of MERS-CoV spike glycoprotein in complex with sialoside attachment receptors. Nat. Struct. Mol. Biol..

[33] Awasthi M., Gulati S., Sarkar D.P., Tiwari S., Kateriya S., Ranjan P., Verma S.K. (2020). The Sialoside-Binding Pocket of SARS-CoV-2 Spike Glycoprotein Structurally Resembles MERS-CoV. Viruses.

[34] Robson B. (2020). Bioinformatics studies on a function of the SARS-CoV-2 spike glycoprotein as the binding of host sialic acid glycans. Comput. Biol. Med..

[35] Tortorici M.A., Walls A.C., Lang Y., Wang C., Li Z., Koerhuis D., Boons G.-J., Bosch B.-J., Rey F.A., De Groot R.J. (2019). Structural basis for human coronavirus attachment to sialic acid receptors. Nat. Struct. Mol. Biol..

[36] Milanetti E., Miotto M., Rienzo L.D., Monti M., Gosti G., Ruocco G. (2020). In-Silico Evidence for Two Receptors Based Strategy of SARS-CoV-2. bioRxiv.

[37] Behloul N., Baha S., Shi R., Meng J. (2020). Role of the GTNGTKR motif in the N-terminal receptor-binding domain of the SARS-CoV-2 spike protein. Virus Res..

[38] Peng G., Xu L., Lin Y.-L., Chen L., Pasquarella J.R., Holmes K.V., Li F. (2012). Crystal Structure of Bovine Coronavirus Spike Protein Lectin Domain. J. Biol. Chem..

[39] Pirone L., Del Gatto A., Di Gaetano S., Saviano M., Capasso D., Zaccaro L., Pedone E. (2020). A Multi-Targeting Approach to Fight SARS-CoV-2 Attachment. Front. Mol. Biosci..

[40] Baker A.N., Richards S.-J., Guy C.S., Congdon T.R., Hasan M., Zwetsloot A.J., Gallo A., Lewandowski J.R., Stansfeld P.J., Straube A. (2020). The SARS-COV-2 Spike Protein Binds Sialic Acids and Enables Rapid Detection in a Lateral Flow Point of Care Diagnostic Device. ACS Central Sci..

[41] Schrödinger, LLC (2019). Schrödinger Release 2019-4: SiteMap.

[42] Bangaru S., Ozorowski G., Turner H.L., Antanasijevic A., Huang D., Wang X., Torres J.L., Diedrich J.K., Tian J.-H., Portnoff A.D. (2020). Structural analysis of full-length SARS-CoV-2 spike protein from an advanced vaccine candidate. Science.

[43] Halgren T.A. (2009). Identifying and Characterizing Binding Sites and Assessing Druggability. J. Chem. Inf. Model..

[44] Fantini J., Di Scala C., Chahinian H., Yahi N. (2020). Structural and molecular modelling studies reveal a new mechanism of action of chloroquine and hydroxychloroquine against SARS-CoV-2 infection. Int. J. Antimicrob. Agents.

[45] Fantini J., Chahinian H., Yahi N. (2020). Synergistic antiviral effect of hydroxychloroquine and azithromycin in combination against SARS-CoV-2: What molecular dynamics studies of virus-host interactions reveal. Int. J. Antimicrob. Agents.

[46] Self W.H., Semler M.W., Leither L.M., Casey J.D., Angus D.C., Brower R.G., Chang S.Y., Collins S.P., Eppensteiner J.C., Filbin M.R. (2020). Effect of Hydroxychloroquine on Clinical Status at 14 Days in Hospitalized Patients With COVID-19: A Randomized Clinical Trial. JAMA.

[47] Abella B.S., Jolkovsky E.L., Biney B.T., Uspal J.E., Hyman M.C., Frank I., Hensley S.E., Gill S., Vogl D.T., Maillard I. (2021). Efficacy and Safety of Hydroxychloroquine vs Placebo for Pre-exposure SARS-CoV-2 Prophylaxis Among Health Care Workers: A Randomized Clinical Trial. JAMA Intern. Med..

[48] Gyselinck I., Janssens W., Verhamme P., Vos R. (2021). Rationale for azithromycin in COVID-19: An overview of existing evidence. BMJ Open Respir. Res..

[49] Caniglia J.L., Guda M.R., Asuthkar S., Tsung A.J., Velpula K.K. (2020). A potential role for Galectin-3 inhibitors in the treatment of COVID-19. PeerJ.

[50] Li F. (2016). Structure, Function, and Evolution of Coronavirus Spike Proteins. Annu. Rev. Virol..

[51] Bertuzzi S., Quintana J.I., Ardá A., Gimeno A., Jiménez-Barbero J. (2020). Targeting Galectins with Glycomimetics. Front. Chem..

[52] Caniglia J.L., Asuthkar S., Tsung A.J., Guda M.R., Velpula K.K. (2020). Immunopathology of galectin-3: An increasingly promising target in COVID-19. F1000Research.

[53] Machala E.A., McSharry B.P., Rouse B.T., Abendroth A., Slobedman B. (2019). Gal power: The diverse roles of galectins in regulating viral infections. J. Gen. Virol..

[54] Sethi A., Sanam S., Munagalasetty S., Jayanthi S., Alvala M. (2020). Understanding the role of galectin inhibitors as potential candidates for SARS-CoV-2 spike protein: In silico studies. RSC Adv..

[55] Hsieh T.-J., Lin H.-Y., Tu Z., Lin T.-C., Wu S.-C., Tseng Y.-Y., Liu F.-T., Hsu S.-T.D., Lin C.-H. (2016). Dual thio-digalactoside-binding modes of human galectins as the structural basis for the design of potent and selective inhibitors. Sci. Rep..

[56] Di Gaetano S., Bedini E., Landolfi A., Pedone E., Pirone L., Saviano M., Traboni S., Capasso D., Iadonisi A. (2019). Synthesis of diglycosylated (di)sulfides and comparative evaluation of their antiproliferative effect against tumor cell lines: A focus on the nature of sugar-recognizing mediators involved. Carbohydr. Res..

[57] Rosa A., Pye V.E., Graham C., Muir L., Seow J., Ng K.W., Cook N.J., Rees-Spear C., Parker E., dos Santos M.S. (2021). SARS-CoV-2 can recruit a haem metabolite to evade antibody immunity. Sci. Adv..

[58] Gutmann C., Takov K., Burnap S.A., Singh B., Ali H., Theofilatos K., Reed E., Hasman M., Nabeebaccus A., Fish M. (2021). SARS-CoV-2 RNAemia and proteomic trajectories inform prognostication in COVID-19 patients admitted to intensive care. Nature communications. Nat. Commun..

